# Induction of cytotoxic effector cells towards cholangiocellular, pancreatic, and colorectal tumor cells by activation of the immune checkpoint CD40/CD40L on dendritic cells

**DOI:** 10.1007/s00262-020-02746-x

**Published:** 2020-11-12

**Authors:** Farsaneh Sadeghlar, Annabelle Vogt, Raphael U. Mohr, Robert Mahn, Katrin van Beekum, Miroslaw Kornek, Tobias J. Weismüller, Vittorio Branchi, Hanno Matthaei, Marieta Toma, I. G. H. Schmidt-Wolf, Jörg C. Kalff, Christian P. Strassburg, Maria A. González-Carmona

**Affiliations:** 1 Department of Internal Medicine I, University Hospital Bonn, University of Bonn, Venusberg-Campus 1, 53105 Bonn, Germany; 2 Department of Visceral Surgery, University Hospital Bonn, Bonn, Germany; 3 Department of Pathology, University Hospital, Bonn, Germany; 4 Center for Integrated Oncology (CIO), Bonn, Germany

**Keywords:** Dendritic cells, Immune checkpoint, Immunotherapy, Cholangiocarcinoma, Pancreatic carcinoma, Hepatocellular carcinoma

## Abstract

**Introduction:**

Gastrointestinal (GI) malignancies, such as cholangiocarcinoma, pancreatic carcinoma, and metastatic colorectal carcinoma, have a poor prognosis and effective therapeutic approaches are still challenging. Checkpoint inhibition with PD-1 or PDL-1 antibodies revealed promising results in different tumor entities; however, only few patients with GI tumors can potentially benefit from PD1/PDL1 inhibiting immunotherapy. Further immunotherapeutic strategies for GI malignancies are urgently needed. The aim of this study was to demonstrate that in vitro activation of the immune checkpoint CD40/CD40L can improve DC action towards bile duct, pancreas, and colorectal carcinoma.

**Methods:**

Human DC were isolated from buffy coats from healthy donors, pulsed with tumor lysates and then transduced with adenoviruses encoding human CD40L (Ad-hCD40L). Using transwell assays, the effects of (m)CD40L on DC immunoactivation compared to (s)CD40L were analyzed. Surface marker and cytokine/chemokine expression were measured by flow cytometry, ELISA and cytokine arrays. Capacity of Ad-hCD40L-transduced DC to induce tumor-specific effector cells was tested using MTT proliferation assay and cytotoxicity assays. Apoptosis induction on tumor cells after culturing with supernatants of Ad-hCD40L-transduced DC was analyzed by flow cytometry.

**Results:**

Ad-hCD40L transduction induced a high expression of (s)CD40L and (m)CD40L on DC and seemed to induce a strong cellular CD40/CD40L interaction among DC, leading to the formation of cell aggregates. Due to the CD40/CD40L interaction, a significant upregulation of DC maturation markers and a Th1-shift on cytokines/chemokines in the supernatant of DC were achieved. Interestingly, a pure Th1-shift was only achieved, when a cellular CD40/CD40L interaction among DC took place. (s)CD40L induced almost no upregulation of maturation markers and rather resulted in a Th2-cytokine expression, such as IL-10. Correspondingly, (m)CD40L-expressing DC led to significant proliferation and stimulation of tumor-specific effector cells with increased cytotoxicity towards pancreatic, bile duct and colorectal tumor cells. Supernatants of Ad-hCD40L-transduced DC could also induce apoptosis in the different tumor cells in vitro.

**Conclusion:**

Stimulation of the immune checkpoint CD40L/CD40 by endogenous expression of (m)CD40L provokes a cellular interaction, which increases the immunomodulatory capacity of DC. A Th1 cytokine/chemokine expression is induced, leading to a significant proliferation and enabling cytotoxicity of effector cells towards human bile duct, pancreatic and colorectal tumor cells. The present data point to the promising approach for DC-based immunotherapy of gastrointestinal malignances by activating the CD40/CD40L immune checkpoint.

**Electronic supplementary material:**

The online version of this article (10.1007/s00262-020-02746-x) contains supplementary material, which is available to authorized users.

## Introduction

Malignancies of the gastrointestinal and pancreaticohepatobiliary system (GI tumors) represent 25% of all malignancies, contributing to a major part of cancer incidence and mortality. Worldwide, CRC is the most frequent entity of GI cancer and accounts for the largest share of GI cancer-related deaths in women; while for men, it is liver cancer [1–3]. Despite the recent advances in diagnosis and therapy [4–10], the prognosis of these tumors in advanced stages remains very poor and effective therapeutic approaches are still challenging.

Recently, immunotherapy of solid tumors using immune checkpoint inhibition, such as PD-1 or PD-L1 antibodies, seems to be a promising new direction of cancer therapy. However, patients with pancreatic cancer, cholangiocarcinoma, and colorectal cancer have shown vast resistance to antibody-mediated checkpoint blockade [11, 12]. Antitumoral activity of PD1- and/or CTLA4-antibodies could only be demonstrated, when GI tumors presented a mismatch repair deficiency (MSI-high) [13–15].

In the context of immunotherapy, the use of dendritic cell (DC) has been shown to be an attractive approach for the treatment of solid tumors in a preclinical setting. DC are professional antigen presenting cells with the ability to initiate and modulate immune response by activating B and T lymphocytes. For that reason, DC might hold an important role in the development of therapeutic immunity against cancer [16–18].

In several phase I and II trials in different types of cancer, DC-based vaccines were well tolerated but could not reach clinical significant antitumoral responses towards GI malignancies [19, 20]. Insufficient activation of DC due to a strong immunosuppressive tumor milieu could explain the lacking effectiveness of DC as solid tumor vaccine.

CD40L is a co-stimulatory molecule usually expressed on activated CD4^+^-T-cells. Through interaction with its receptor CD40, expressed on DC, cytotoxic T cells can be primed [21, 22]. In own preclinical studies, we demonstrated that transduction with Ad-CD40L increased the stimulatory capacity of DCs significantly and activated both acquired and innate immune response towards HCC in vitro and in vivo [23, 24].

In this study, adenoviral-mediated endogenous expression of (m) CD40L activated the CD40/CD40L checkpoint and improved dendritic cells-mediated cytotoxicity of effector cells against cholangiocarcinoma, pancreas carcinoma and colorectal carcinoma in vitro.

## Materials and methods

### Cell lines

The human embryonic cell line 911 was used for the amplification of the E1- and E3-deleted adenoviral vectors [25]. The extrahepatic bile duct cancer cell line EGI-1 (DSMZ ACC 385), the gallbladder cancer cell line MZ-ChA-2 [26] and the colorectal cell lines, HT-29 (ATCC HTB-38) and LoVo (ATCC CCL-229), were cultured in DMEM, supplemented with 10% fetal calf serum (FCS), (Biochrom, Berlin, Germany), 100 units/ml of penicillin and 100 µg/ml of streptomycin (Sigma, Deisenhofen, Germany) at 37 °C at 5% CO_2_ atmosphere. Capan-1 (ATCC HTB-79) and Dan-G (German DSMZ ACC 249) are human pancreatic cell lines. TFK-1(German DSMZ ACC 344) is a human bile duct carcinoma cell line. All three cell lines were cultured in RPMI1640 with 10% FCS, 100 units/ml of penicillin and 100 µg/ml of streptomycin.

### Plasmids and adenoviral vectors

The hCD40L-encoding plasmid pORF-hCD40LG was purchased from Invivogen (Toulouse, France). The hCD40L gene was cut out and cloned into pShuttle-CMV to generate pShuttleCMV–hCD40L, recombined with the adenoviral backbone plasmid pAdEasy-1 by homologous recombination and transfected into 911 cells to generate hCD40L-encoding E1-deleted Ad (Ad-hCD40L) as described previously (Stratagene, La Jolla, USA) [27]. Further E1- and E3-deleted adenoviral vectors were used: Ad-Mock (without transgene, served as control) and Ad-GFP (encoding for green fluorescence protein).

### Generation of DC

Peripheral blood mononuclear cells (PBMC) were isolated from buffy coat from healthy donors after informed consent as described previously [27]. Blood was drawn according to the instruction of our local ethics committee. To receive monocyte-derived DC, cells were separated through two density gradients using ficoll (Lymphoprep-Nycomed, Norway) and OptiPrep Density Gradient Medium (Sigma-Aldrich, München, Germany). Monocyte-derived DC were cultured in RPMI1640 medium supplemented with 10% heat-inactivated, autologous serum and 750 U/ml GM-CSF and 500 U/ml IL-4 (Immunotools, Friesoythe, Germany). The medium was replaced on day + 4 after the DC generation.

### Adenoviral transduction of DC

To pulse DC with tumor-associated antigens, tumor cell lysates were prepared through repeated freeze and thaw cycles. After centrifugation, protein concentration was determined in the supernatant using the BCA protein detection kit (Thermo, Bonn, Germany) according to the manufacturer’s instructions. Five days after their generation, DC were pulsed with the different tumor lysates (100 µg/ml).

On day + 6 after their generation, DC were adenovirally transduced with Ad-CD40L at MOI 100. DC were  transduced in phosphate-buffered saline (PBS) (PAA) with 2% heat-inactivated autologous serum for 2 h at 37 °C.

### Flow cytometry

DC were immunophenotyped two days after their adenoviral transduction (day + 8) using flow cytometry. Monoclonal antibodies specific for human CD11c, CD83, CD86, CD154, HLA-DR (Pharmingen, Hamburg, Germany), CCR7 (R&D Systems, Wiesbaden, Germany), CD40, CD80 (eBioscience, Frankfurt, Germany) together with their appropriate isotype control were used. Cells were stained for 15 min on ice with the different antibodies, washed twice with PBS and were then resuspended in PBS. Prior to analysis, 4′-6-Diamidino-2-phenylindole (DAPI) was added to each sample to differentiate between viable and dead cells. 30.000 events were analyzed for every sample. Flow cytometry was performed using a FACSCanto II with Diva software (BD Bioscience) and FlowJo7.2.2 (Tree Star Inc. Ashland, USA) for analysis.

To determine the levels of CD40 on the different tumor cells, 1 × 10^6^ cells were stained for 30 min with anti-CD40 antibody or the appropriate isotype control and analyzed by flow cytometry as described above.

### Determination of cytokine/chemokine expression levels

Cytokine and chemokine expression was analyzed two days after adenoviral transduction in the supernatant of DC as well as in the supernatant of CIK cells after coculturing for four days with transduced DC using commercial ELISA kits: human TNFalpha DuoSet (R&D Systems, Wiesbaden, Germany; 88-7126-22, DY210-05, DY008), human Interferon-gamma, eBiosciences (San Diego, USA, 14-7318-67) and human IL-10 from eBiosciences (San Diego, USA, 14-7108-67),according to the manufacturer’s instructions. For multianalyte cytokine detection, using the cytokine multiplex assay kit, the Cytokine Antibody Arrays (R&D Systems, Minneapolis, USA) were used. The sample mixtures were incubated with the cytokine array membrane and bound by capture antibody on the membrane, if present. After a wash step to remove unbound material, Streptavidin–HRP and chemiluminescent detection reagents were added. Light was produced at each spot in proportion to the amount of cytokine bound. The cytokine array was analyzed by chemiluminescence. Amounts of cytokine were semiquantified and compared using a densitometry software.

### Blocking of CD40/CD40L interaction of DC

DC were transduced with Ad-hCD40L on day + 6 as described above. Different concentrations (2.5, 12.5 and 25 µg) of a blocking antibody for CD40L or the isotype control antibody (R&D Systems, Wiesbaden, Germany) were added to the cells. After 2 days of incubation, IL-12 levels were analyzed.

### Transwell assay

To test the influence of Ad-hCD40L-transduced DC on further DC, transwell experiments were performed. For this purpose, 1 × 10^6^ Ad-Mock-DC were seeded in the lower chamber of a 1 µm transwell plate (Corning Costar, Cambridge, MA) after their transduction. Ad-hCD40L-transduced DC were added in the upper chamber. After 2 days of incubation, cells of the upper chamber were discarded and cells of the lower chamber were extensively washed and incubated for another 48 h. Supernatant of DC was then analyzed for cytokine expression using ELISA technique and DC were analyzed for surface marker expression using flow cytometry.

### Detection of apoptosis

To test for effects on apoptosis induction, tumor cells were incubated with conditioned supernatant retained of DC, which were previously transduced with Ad-Mock and Ad-hCD40L for 48 h or non-transduced. Briefly, cells were harvested and fixed with 70% ethanol. Cells were incubated with DNA extraction buffer before their DNA was stained with propidium iodide. The subG1-fraction was analyzed using flow cytometry.

### Generation of cytokine-induced killer (CIK) cells and coculture with DC

Human CIK cells were generated as previously described [27]. Briefly, non-adherent Ficoll-separated PBMC were cultured with 1000U/ml IFN-γ (Immunotools, Friesoythe, Germany). One day later, 50 ng/ml anti-CD3 antibody (ebiosciences, Frankfurt, Germany), 100U/ml IL-1β and 300U/ml IL-2 (immunotools, Friesoythe, Germany) were added. CIK cells were cocultured with tumor cell lysate-pulsed and adenoviral-transduced DC at a ratio of 5:1. After four days of coculture, the cytotoxic response of CIK cells towards Lovo, DanG or EgI-1 cells was analyzed.

### MTT test

Proliferation of effector cells (CIK cells) after coculturing with CD40L-expressing DC was assessed using the MTT test (3-(4,5-Dimethylthiazol-2-yl)-2,5-Diphenyltetrazolium Bromide). MTT tests were performed with Tetrazolum. This indicator can enter the cell and is then reduced to Formazan, which can be photometrical detected at 550 nm and correlates with the cell proliferation. Therefore, CIK cells were cocultured with DC in 96-well plates in a ratio of 5:1 over a time period of four days. MTT tests were performed on day 0, 2 and 4. For analysis, CIK cells were dissolved in 100 µl MTT solution. After an incubation time of 45 min, cells were centrifuged, the supernatants removed and MTT was solubilized with 100 µl DMSO. The optical density was measured colorimetrically at 550 nm after 5 min of incubation.

### Cytotoxicity assay

Cytotoxicity of tumor cells by CIK cells, which were cocultured with CD40L-expressing DC towards different tumor cells (EgI-1, LoVo, DanG, TFK-1) was determined using the Cytotox-One Homogeneous Membrane Integrity Assay (Promega, Mannheim, Germany), following the manufacturer’s instructions. This assay is based on the release of lactate dehydrogenase (LDH) from damaged cells. Fluorescence signal was measured with a GloMax Multi ELISA reader (Promega, Mannheim, Germany). The different target cells were incubated with the CIK effector cells at different ratios for 4 h. Maximum release was obtained by incubating target cells with lysis buffer. Target cells with effector cells at different ratios served as negative control (spontaneous release). Specific lysis was calculated as follows: Percent cytotoxicity = (experimental release − spontaneous release)/(maximal release − spontaneous release).

### Immunohistochemistry

CD40 expression on human tumor samples was evaluated using a monoclonal anti human CD40 antibody (AMAB90905, Sigma Aldrich, Taufkirchen, Germany) by immunohistochemistry in our Institute of Pathology. CD40 expression was evaluated based on the combined positive score (CPS). CPS was calculated by the total number of CD40-positive cells including tumor cells, lymphocytes and macrophage in relation to the total number of viable tumor cells (multiplying by 100).

### Statistical analysis

Data are presented as means with standard errors of the mean (SEM). Statistically significant difference between experimental groups was analyzed using the paired *T* test. A *p* value of less than 0.05 was considered significant.

## Results

### CD40L transgene expression was highly expressed in human DC after adenoviral transduction with Ad-hCD40L

**Fig. 1:**
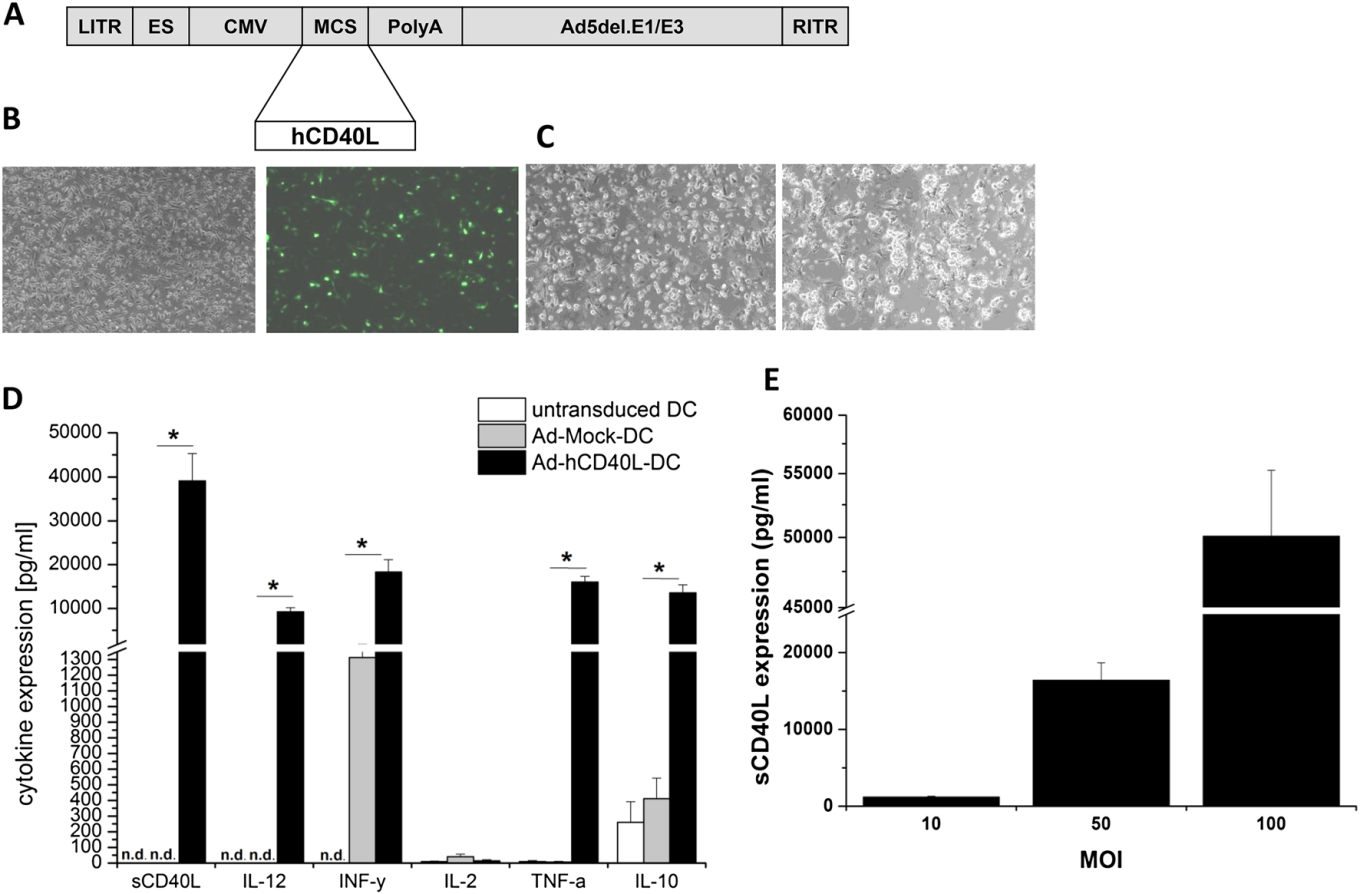
Characterization and immunstimulation of Ad-hCD40L-transduced DC. **a** Map of Ad-hCD40L. *LITR* left-inverted terminal repeat, *ES* encapsidation signal, *CMV* cytomegalovirus immediate early promoter, *MCS* multiple cloning site, *PolyA* polyadenylation signal, *Ad5del.E1/E3* human adenovirus type 5 sequence with deletion of E1/E3 genes, *RITR* right-inverted terminal repeat. **b** Light (left) and fluorescence (right) microscopy of DC 48 h after transduction with Ad-GFP (magnification × 10). **c** Light microscopy of DC 48 h after transduction with Ad-Mock (left) or Ad-hCD40L (right). Ad-hCD40L-transduced DC form cell aggregates (magnification × 10). **d** Cytokine amounts in the supernatant of DC 48 h after adenoviral transduction with Ad-hCD40L, Ad-Mock or non-transduced measured by ELISA. (*p < 0.05), n.d. = non-detectable. Data represent means ± SEM of six different experiments. **e** Expression of sCD40L 48 h after transduction of DC with Ad-hCD40L with different MOIs, (*n* = 4–5). Data represent means ± SEM of three different experiments

To investigate the transduction efficiency and transgene expression of DC, hDC were transduced with Ad-GFP and with Ad-hCD40L. 48 h later, the expression of soluble (s)CD40L was confirmed by ELISA (Fig. 1a/e). Approximately 30–40% of DC have expressed GFP (MOI 100), (Fig. 1b). Interestingly, transduction of DC with Ad-hCD40L led to aggregate formation proving the interaction between CD40L and CD40 (Fig. 1c). Ad-hCD40L-transduced DC secreted high levels of sCD40L as well (39.116 ± 6155 pg/ml) (Fig. 1d/e).

**Fig. 2:**
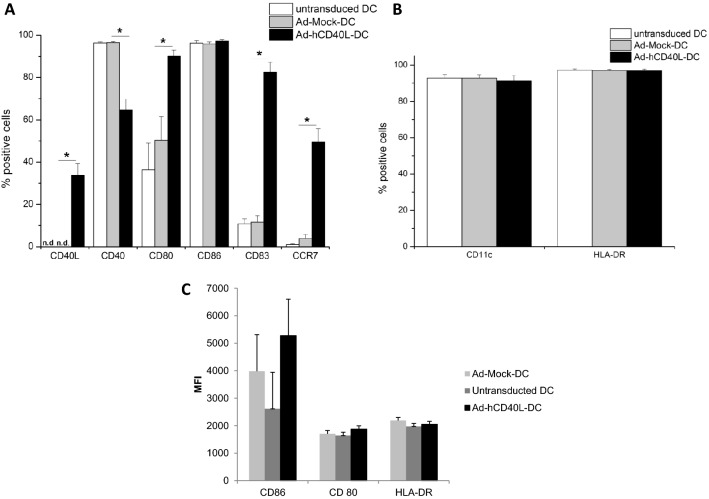
**a**, **b** Flow cytometry of surface and maturation markers of DC 48 h after adenoviral transduction with Ad-hCD40L, Ad-Mock or non-transduced. After pre-gating of CD11c-positive cells, the percentage of positive cells was analyzed. *p < 0.05, *n.d.* non-detectable. Data represent means ± SEM of six different experiments. *n* = 6–10

Correspondingly, Ad-hCD40L-transduced DC expressed 33.7 ± 5.5% of membrane-bound (m) CD40L, whereas no CD40L could be detected in the control-transduced DC (Fig. 2a).

Flow cytometric analysis showed no significant change in positive cells profile of DC surface markers (CD11c and HLA-DR) between ad-hCD40L-DC and non-transduced DC or AD-Mock-DC (Fig. 2b).

### Expression of surface costimulatory markers was highly increased on human DC due to adenoviral transduction with Ad-hCD40L

To further investigate whether there is an immunostimularory effect after transduction of DC with Ad-hCD40L, flow cytometry analysis was performed to characterize the phenotype of DC.

For CD80, the number of positive Ad-hCD40L-transduced DC was significantly (*p* = 0.0198) higher (90.1 ± 2.9%) compared to the number of positive CD80 + Ad-Mock DC (50.3 ± 11.3%). A significantly (*p* < 0.0001) higher CD83 + Ad-hCD40L-transduced DC could be observed compared to controls. The number of CCR7 + Ad-hCD40L-DC, was also significantly (*p* = 0.0001) increased compared to Ad-Mock DC (Fig. 2a). This CD surface marker is important for the migration of DC. Thus, transduction with Ad-hCD40L shows effective immune activation of DC compared to Ad-Mock and untransduced DC. Interestingly, the number of CD40 + Ad-hCD40L-transduced DC was significantly (*p* = 0.0003) reduced (1.5-fold) compared to Ad-Mock DC or untransduced DC (Fig. 2a). This effect could be possibly caused by the strong interaction between DC due to the CD40/CD40L interaction, blocking the CD40 receptor for binding with the fluorescence antibody. All populations of untranduced-, Ad-Mock- and Ad-hCD40L-DC presented about 90% CD11+ and HLA-DR+ cells and did not differ from each other (Fig. 2b).

For CD86, MFI evaluation revealed a significantly higher expression on Ad-hCD40L-transduced DC compared to untransduced DC (Ad-Mock DC). MFI analysis showed no significant change in the expression profile of DC surface markers CD80 and HLA-DR between Ad-hCD40L-DC, Ad-Mock-DC and untransduced DC (Fig. 2c).

### Increased expression of Th1 and Th2 cytokines/chemokines on human DC due to adenoviral transduction with Ad-hCD40L

To elucidate the effects of Ad-hCD40L transduction on cytokine/chemokine expression, we analyzed the supernatants of the DC by ELISA and cytokine array.

As shown in ESM Fig. 1a in the semiquantitative cytokine array, the expression of MIP-1a and MIP-1ß was significantly (*p* = 0.0042, *p* = 0.0008, respectively) enhanced in Ad-hCD40L-transduced DC compared to Ad-Mock DC. Furthermore, significantly higher levels of proinflammatory cytokines such as IL-1β (*p* = 0.0117), IL-6 (*p* < 0.0001) or IL-8 (*p* = 0.0103) were measured in the supernatant of Ad-hCD40L-transduced DC; whereas, almost no expression was found in Ad-Mock-transduced DC (ESM Fig. 1b). Further cytokines and chemokines such as IL-17, IL-23, IL-27, IL-32a, IP-10 as well as I-TAC were all significantly (*p* < 0.05) upregulated after transduction of DC with Ad-hCD40L compared to Ad-Mock (ESM Fig. 1c). As shown in ESM Fig. 1d, a significant increase for Rantes, SDF-1, sTREM-1, sICAM-1, GROa as well as for G-CSF could be detected for Ad-hCD40L DC.

**Fig. 3:**
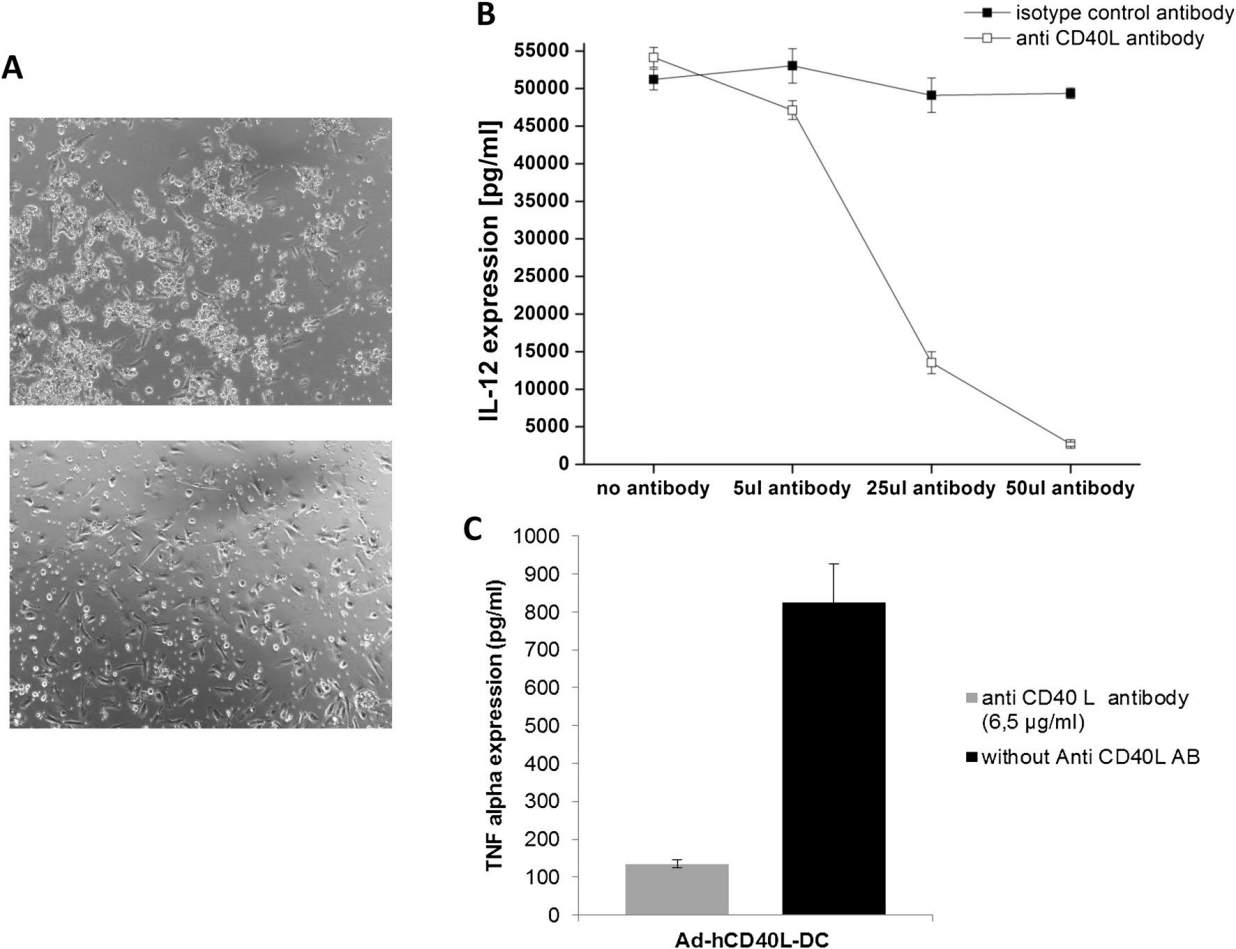
Blocking of CD40/CD40L interaction.** a** IL-12 expression after blocking of CD40/CD40L interaction of Ad-hCD40L transduced DC with an anti-CD40L antibody or isotype control antibody. The blocking antibody was added to the supernatant in the indicated concentrations directly after transduction of DC for 2 days. Data represent means ± SEM of four different experiments. (*p < 0.05). **b** Light microscopy of DC 48 h after transduction with Ad-hCD40L (left) and after adding of the blocking antibody (right). No aggregate formation could be detected after culturing with the blocking antibody (magnification × 10). **c **TNF-alpha expression in supernatant of Ad-hCD40L-DC after adding the blocking antibody

In quantitative ELISA assays, the expression of high amounts of IL-12 (9217 ± 963.9 pg/ml) in hCD40L-expressing DC was confirmed, whereas no IL12-expression could be detected in the supernatant of Ad-Mock or untransduced DC. Blocking the CD40/CD40L interaction could significantly block the IL-12 production, clearly indicating that the immune checkpoint CD40/CD40L was totally responsible for the IL-12 expression observed. The reduced levels of IL-12 in the supernatant of transduced DC were dependent on the concentration of the blocking antibody; whereas, the IL-12 secretion of DC incubated with the control antibody remains unaffected (Fig. 3b). The blocking of CD40L led to a reduced IL-12 expression. Moreover, the aggregate formation of Ad-hCD40L-transduced DC was not visible 48 h after addition of blocking antibody compared to DC incubated with the control antibody (Fig. 3a).

Moreover, expression levels of further Th1-cytokines such as IFNgamma and TNFalpha were also significantly increased (*p* = 0.0003, *p* ≤ 0.0001, respectively) only in Ad-hCD40L-transduced DC, confirming a strong Th1 shift of cytokine expression due to the expression of CD40L.

**Fig. 4:**
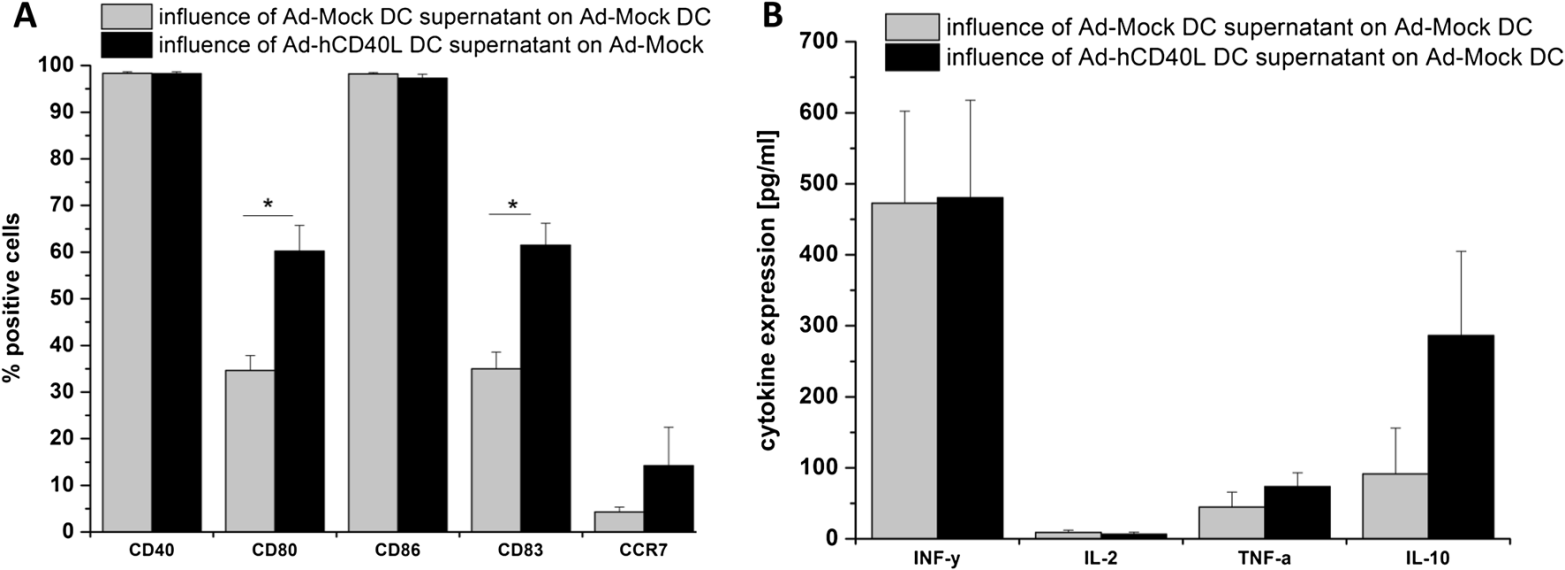
Influence of sCD40L on Ad-Mock DC. **a** Flow cytometry of surface and maturation markers of DC 48 h after 2 days of incubation of Ad-hCD40L DC with Ad-Mock DC in a transwell assay. The influence of Ad-hCD40L DC supernatant on surface marker expression of Ad-Mock DC was analyzed. After pre-gating of CD11c-positive cells, the percentage of positive cells was analyzed. *p < 0.05, *n.d.* non-detectable. Data represent means ± SEM of 3–4 different experiments. **b** Cytokine amounts in the supernatant of DC 48 h after 2 days of incubation of Ad-hCD40L DC with Ad-Mock DC in a transwell assay. The influence of Ad-hCD40L DC supernatant on the cytokine expression of Ad-Mock DC was analyzed by ELISA. (*p < 0.05), *n.d.* non-detectable. Data represent means ± SEM of 3–4 different experiments

Expression of TNF-alpha could also be inhibited (84%) after adding of anti-CD40L antibody to the Ad-hCD40L DC culture medium (Fig. 3c). However, Ad-hCD40L-transduced DC also appeared to induce the expression of Th2-cytokines as well, such as IL10, which was expressed significantly (*p* < 0.0001) higher (13,601 ± 1813) in the supernatant of CD40L-expressing DC compared to Ad-Mock (411 ± 132.3 pg/ml) or untransduced DC (260.2 ± 131.3 pg/ml) DC (Fig. 4a).

### Influence of Ad-hCD40L-transduced DC on neighboring DC

**Fig. 5:**
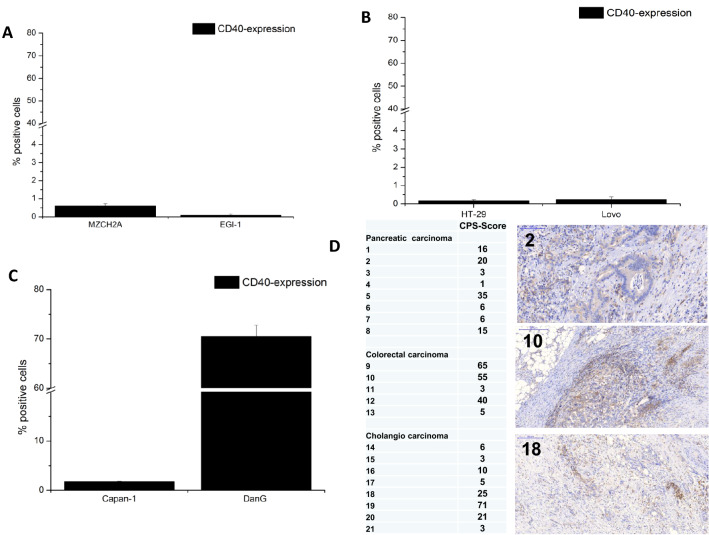
CD40 expression on tumor cells. **a–c** Flow cytometry analysis of CD40 expression on CCC (**a**), (CRC (**b**) or pancreatic cancer (**c**) cell lines. Data represent means ± SEM of three different experiments. **d** Left: table with results of CD40 CPS (combined positive score) of 21 patients with pancreatic, colorectal and bile duct carcinoma. Right: Microscopic findings for remnant Gastrointestinal malignancies of three representative samples after immunohistochemical staining of CD40 (× 20). Patient number 2 with pancreatic carcinoma, Patient number 10 with colorectal carcinoma and patient number 18 with bile duct carcinoma

Since we observed highly expressed amounts of Th1 cytokines and Th2 cytokines in the supernatant of hCD40L-transudced DC, it was intriguing to investigate whether the supernatant of those dendritic cells has immunostimulatory effects due to the expression of sCD40L and Th1-cytokines or inhibitory effects due to the expression of immunosuppressive cytokines, such as IL10 on neighbor dendritic cells Fig. 4b. For this purpose, Ad-Mock DC were seeded in the lower chamber of a transwell and were cultured with Ad-hCD40L transduced DC, which were located in the upper chamber. As shown in Fig. 4a, b, 5a, the surface marker expression of Ad-Mock DC was influenced by the Ad-hCD40L DC supernatant. The number of CD80+ and CD83+ cells was significantly higher compared to Ad-Mock DC incubated with the supernatant of Ad-Mock DC (*p* = 0.0024, *p* = 0.0003, respectively). However, reached levels of expression of Fig. 2. Moreover, for CCR7 expression, a slight but not significant increase could be detected Fig 4a.

Regarding cytokine expression, we unexpectedly found no increased IFN gamma and IL-2 expression. Th2-cytokines such as, TNF-alpha and IL-10, were even slightly increased on DC by culturing with Ad-hCD40L DC supernatant, suggesting that the supernatant was not sufficient to induce a strong immunostimulatory effect on DC as observed for cellular interaction of CD40L/CD40 on DC as shown in Fig. 1.

### Ad-hCD40L-transduced DC increased the proliferation of effector cells (CIK cells)

**Fig. 6:**
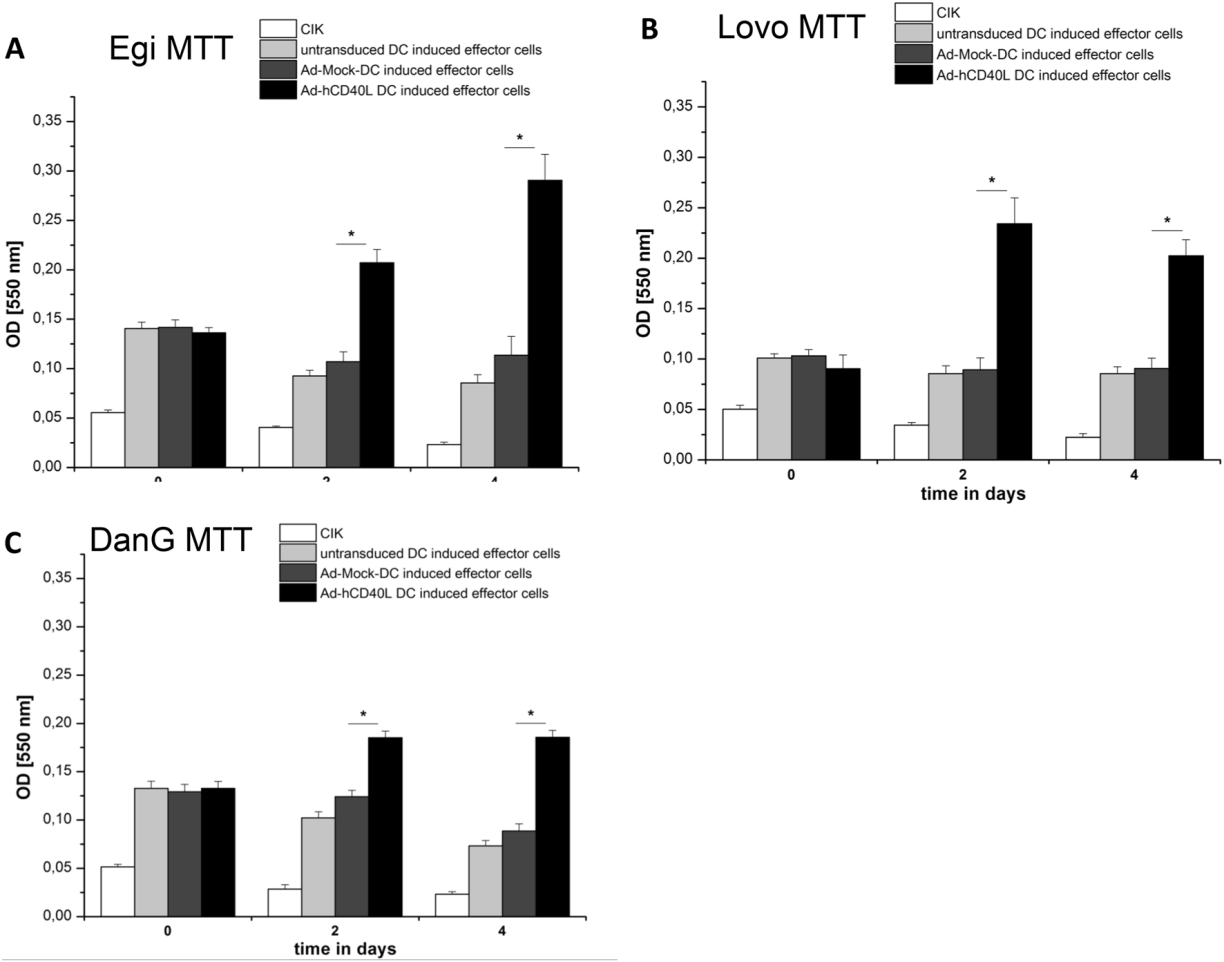
Proliferation of effectors cells after stimulation with Ad-hCD40L-DC. **a**–**c** Measurement of cell proliferation of effector cells (CIK) by MTT test on days 0, 2 and 4 of coculturing with Ad-hCD40L-, Ad-Mock- or non-transduced DC. DC were pulsed with tumor-lysate from EgI-1-, Lovo- and DanG-cells before coculture*.* Data represent means ± SEM of four different experiments. (*p < 0.05)

We now explored whether Ad-hCD40L-transduced DC were capable to stimulate proliferation of CIK cells. Therefore, CIK cells were co-incubated with either Ad-hCD40L or Ad-Mock or untransduced DC, which were pulsed before with tumor cell lysate from EgI-1, DanG or Lovo cells, for 4 days. Cell proliferation was measured using MTT assay. Proliferation of CIK cells was significantly increased already two days of co-incubation with Ad-hCD40L DC but not when co-incubated with Ad-Mock-DC or untransduced DC. Another two days later of co-incubation (day 4), the proliferation of Ad-hCD40L-DC-induced CIK cells was even higher than on day 2 and significantly (*p* < 0.05) increased (Fig. 6a–c). This effect was irrespective of which tumor cell lysate was used for the pulsing of the DC. These data demonstrate clearly that Ad-hCD40L DC are able to stimulate CIK cell proliferation.

### Effect of Ad-hCD40L-DC on cytokine expression from effector cells (CIK cells)

After showing that Ad-hCD40L DC increased proliferation of CIK cells, we further investigated whether Ad-hCD40L DC also affect cytokine expression of CIK cells. Therefore, we analyzed the supernatant after 4 days of coculture. As shown in ESM Fig. 2a, the expression of the Th1 cytokine IFN gamma was significantly increased (*p* < 0.05) in the supernatant of CIK cells cocultured with Ad-hCD40L-transduced DC irrespective of which tumor cell lysate was used for the pulsing of the DC. By contrast, cocultivation of CIK cells with Ad-hCD40L DC showed no alteration for IL-2 expression (ESM Fig. 2b). As shown in ESM Fig. 2c, we found increased levels of IL-10 for CIK cells alone; while, coculturing CIK cells with untransduced, Ad-Mock-transduced or Ad-hCD40L-transduced DC led to a decreased IL-10 production. For Lovo-pulsed Ad-hCD40L-transduced DC, an increase of IL-10 (threefold) compared to cocultures with Ad-Mock DC could be detected (*p* < 0.0001).

Ad-hCD40L DC, which were pulsed with EGI-1 cell lysates did not influence the IL-4 expression of CIK cells compared to controls; whereas for Lovo- and DanG-pulsed Ad-hCD40L-DC, a slight decrease of IL-4 could be detected compared to Ad-Mock-DC coculturing (ESM Fig. 2d).

Additionally VEGF, which is known to be the main promoter of angiogenenesis was analyzed in the supernatant after coculturing DC with CIK cells. For DanG-pulsed DC, only low levels of VEGF could be measured in the supernatant and no significant increase could be detected after Ad-hCD40L DC coculturing (ESM Fig. 2e).

By contrast, cocultivation of EgI-1-and Lovo-pulsed Ad-hCD40L DC enhanced VEGF expression significantly (*p* = 0.0421, *p* = 0.0297, respectively) compared to Ad-Mock DC.

### Increased cytotoxicity of CIK cells towards pancreatic, cholangiocarcinoma and colorectal tumor cells after coculture with CD40L-expressing DC

After detecting an Ad-hCD40L DC-dependent increase in proliferation and changes in the cytokine expression of CIK cells, it was interesting to investigate whether an enhanced cytotoxic potential towards the different tumor cell lines could be found. Therefore, DC were pulsed with either EgI-1, Lovo, DanG and TFK-1 tumor cell lysate on day + 5 and transduced with Ad-Mock or Ad-hCD40L on day + 6. CIK cells were then cocultured with the different DC for four days. Specific lysis of CIK cells towards, EgI-1, Lovo or DanG cells was investigated using LDH-based cytotoxicity assay.

For all analyzed GI cell lines, specific lysis of tumor cells was significantly higher (*p* < 0.05) when CIK cells were cocultivated with Ad-hCD40L-transduced DC than with Ad-Mock DC or untransduced DC (*p* < 0.05). The best lysis could be yield when effector:target ratio was 80:1. (ESM Fig. 3). Thus, coculturing CIK cells with tumor-lysate-pulsed Ad-hCD40L-DC improves significantly the cytotoxic potential.

### Th1-shifted supernatant of Ad-hCD40L-transduced DC induced tumor cell apoptosis

A possible mechanism for the induction of apoptosis in tumor cells is the interaction between sCD40L and CD40 expressed on tumor cells. Therefore, we first analyzed the levels of CD40 expression on all tumor cell lines. Only the pancreatic cell line DanG showed a high level of CD40+ cells (70.5 ± 2.3%). For all other cell lines, very low CD40 levels (< 2%) could be measured (Fig. 5a–c). We also examined CD40 expression in human tissues (Fig. 5), showing that more than 50% of tumor samples of different patients suffering from pancreatic, colorectal or bile duct cancer, have a positive CPS for CD40 of more than 10%.

To examine if the supernatant of Ad-hCD40L DC is able to induce apoptosis in the different tumor cell lines, cells were cultured with the supernatant of Ad-hCD40L-transduced DC or control DC and subG1 fraction was determined as sign of apoptosis using flow cytometry.

**Fig. 7:**
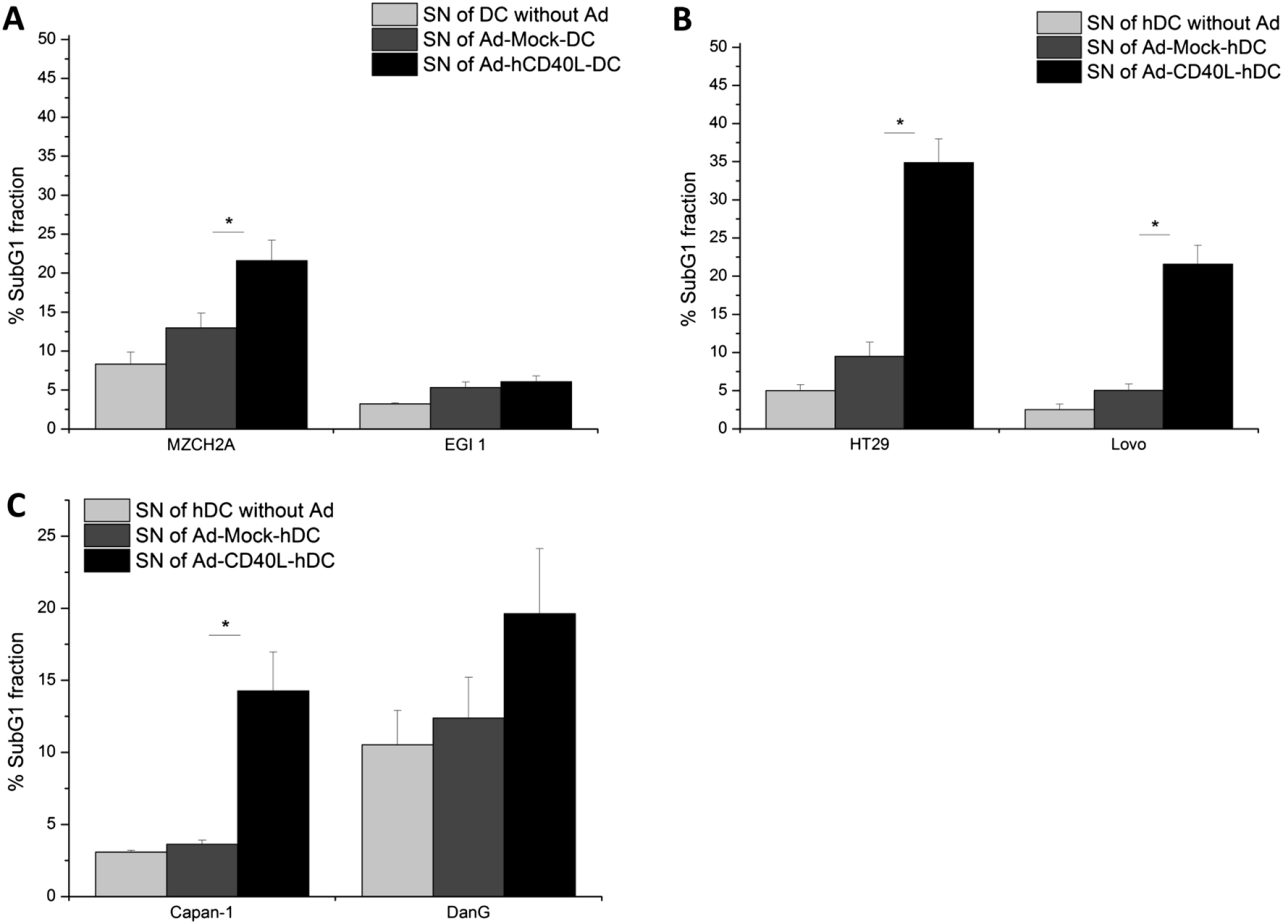
Apoptotic effects of Ad-hCD40L-DC supernatant on tumor cells. **a**–**c** Determination of SubG1 fraction CCC (**a**), CRC (**b**) or pancreatic cancer (**c**) cell lines after incubation with supernatant (SN) of Ad-hCD40L, Ad-Mock or non-transduced DC. Data represent means ± SEM of six different experiments. (*p < 0.05)

Interestingly, we found significant apoptosis induction in MZCH2A cells (*p* = 0.0310), in both colorectal cell lines ((HT-29 and Lovo, (*p* < 0.0001, *p* = 0.0364)) as well as in the pancreatic cell line Capan-1 (*p* = 0.0161), (Fig. 7a–c) after culturing with Ad-hCD40L-DC supernatant compared to Ad-Mock-DC supernatant although these cell lines expressed only low amounts of CD40. For EGI-1 cells which also expressed only low CD40, no induction of apoptosis was found after culturing with Ad-hCD40L DC supernatant. DanG cells with the highest expression of CD40 showed only a slight increase in apoptosis rate after culturing with Ad-hCD40L-DC supernatant compared to Ad-Mock-DC supernatant (Fig. 7c). Thus, no correlation could be detected between CD40 expression and apoptosis induction.

## Discussion

In this study, we showed that Ad-hCD40L-transduced DC induce a CD40/CD40L-dependent cellular interaction among DC, leading to a significant upregulation of maturation markers and a Th1-shift of cytokine/chemokine expression in the supernatant of DC. This effect could be attributed more to the adenoviral-mediated expression of membrane-bound CD40L on DC, than to soluble CD40L. (m)CD40L-activated DC were able to stimulate Th1-cytokines, effector cells proliferation, and cytotoxicity towards colorectal, pancreatic, and bile duct tumor cells in vitro. Thus, activation of the immune checkpoint CD40/CD40L may represent a promising approach to improve DC and CIK cell-based immunotherapy of gastrointestinal malignances.

Cholangiocarcinoma, pancreatic carcinoma and metastatic colorectal carcinoma have a poor prognosis and effective therapeutic approaches are still challenging. Despite the promising results of PD-1/PDL-1 antibody-based immunotherapy in different tumor entities, only few patients with GI tumors can potentially benefit from that approach. Further immunotherapeutic strategies for these patients are warranted.

In a preclinical setting, DC were demonstrated to be a potential approach for the treatment of solid tumors [16, 18]. Although DC-based immunotherapy was shown to be feasible in several phase I and II trials, the clinical benefit was disappointing [19, 20]. A major challenge for a successful DC-based immunotherapy is the achievement of a sufficient immunoactivation of DC that consecutively is able to initiate and boost a strong Th-1 immune response towards tumor cells. In this context, it has already been demonstrated that DC maturation and IL-12 production represent two key factors for a successful antitumoral immune response and progression-free survival [28, 29].

Activating the immune checkpoint CD40/CD40L leads to DC maturation, stimulates Th1 immunity, and enables M2 to M1 macrophage differentiation [24]. Adenoviral vectors increase transgene expression of DC [30, 31]. We used CD40L-coding adenoviral vectors to achieve a high expression of CD40L (membrane bound and soluble). This induced a cellular interaction with aggregate formation among DC and led to a strong immunostimulation by IL-12 induction. Furthermore, a high quantity of costimulatory molecules was reached, such as CD83, an important marker for maturation of human DC, as well as CCR7, which is necessary for lymphatic migration [32]. The number of CD40+ cells appeared to be reduced, which may be explained by a reduced binding of CD40 antibody detection in the flow cytometry because of the competitive cellular CD40/CD40L interaction.

Regarding soluble levels of cytokine/chemokine expression on DC supernatant, we could observe a strong Th1 shift with significant higher secretion not only of IFN gamma, IL12, IL-23, RANTES, G-CSF or TNF-alpha, but also of Th2-cytokines, such as IL-10 and IL6 and even IL-17 and IL17E, which indicates the presence of immature or semimature DC [33]. The effect of (s) CD40L seemed to induce mainly Th2 cytokines/chemokines, whereas an endogenous expression of (m)CD40L induced a clear Th1 shift as observed in our transwell experiments and by blocking (s)CD40L.

Clinical trials using recombinant (s) CD40L or anti-CD40 agonistic antibodies have shown promising results in several malignancies, including pancreatic cancer [34, 35]. However, systemic application of (s)CD40L or anti-CD40 may cause high toxicity by inducing a generalized proinflammatory reaction. Moreover, (s)CD40L may even trigger carcinogenesis by activating inflammatory pathways. In patients with different malignancies, including pancreatic and colorectal cancer, higher levels of circulating sCD40L have been observed [36].

Recently, an oncolytic adenovirus expressing membrane-bound CD40L was evaluated in a preclinical setting as a treatment for pancreatic cancer, leading to tumor control via stimulation of human myeloid cells and T-cell responses [24].

In our study, expression of (m)CD40L was targeted into DC ex vivo. These most activated DC were able to induce a Th1 cytokine expression on effector cells, proliferate, and induce tumor-specific cytotoxicity towards colorectal carcinoma, cholangiocarcinoma and pancreatic carcinoma. Interestingly, we observed in 50% of tumor samples of patients with colorectal, bile duct and pancreatic carcinoma a strong expression of CD40.

However, the observed effect in this study was not related to a positive expression of CD40 by tumor cells. Probably, other factors such as cytokine expression of IFN gamma or TNF-alpha may be involved in inducing tumor cell apoptosis.

In conclusion, membrane-bound CD40L-expressing DC resulted in highly activated DC and Th1-shifted cytokine/chemokine expression which stimulates effector cells towards colorectal, bile duct and pancreatic tumor cells. Despite the promising data presented in this work, limitations derived from in vitro experiments have to be taken into account. Further translational research and phase I trials are urgently needed to evaluate the clinical application as vaccines or intratumoral injections.

### Electronic supplementary material

Below is the link to the electronic supplementary material.

**Figure 1** Cytokine/chemokine expression pattern after Ad-hCD40L transduction of DC using cytocine array.(A-D) Amounts of cytokine- and chemokine expression (pg/ml) in the supernatant of DC 48 hours after adenoviral transduction with Ad-hCD40L or Ad-Mock. n.d.=non detectable. Data represent means+/-SEM of four different experiments. (*=p<0.05). **Figure 2** Cytokine expression in the supernatant of co-cultured effector cells with transduced DC.(A-E) Amounts of IFNγ (A), IL-2 (B), IL-10 (C), IL-4 (D) and VEGF (E) measured by ELISA in the supernatant of CIK cells cocultured with Ad-hCD40L-, Ad-Mock- or non-transduced DC for four days. DC were pulsed with tumor-lysate from -, EgI-1-, LoVo- and DanG-cells before coculture. Data represent means+/-SEM of three different experiments. (*=p<0.05). **Figure 3** Enhanced cytotoxicity of effector (CIK) cells after cocultering with Ad-hCD40L transduced DC.Specific cytotoxicity of autologous effector cells (CIK-cells) towards, EgI-1-, Lovo- and DanG cells after coculture with Ad-hCD40L-, Ad-Mock- or non-transduced DC. Data represent means+/-SEM of three-four different experiments. (*=p<0.05). (PPT 1110 kb)

## Abbreviations

Ad-GFP: Adenovirus containing green fluorescent protein
Ad-hCD40L: Adenoviruses encoding human CD40 ligand
CD40L: Cluster of differentiation 40 ligand
CIK: Cytokine-induced killer cells
CPS: Combined positive score
CRC: Colorectal cancer
CTLA-4: Cytotoxic T-lymphocyte-associated protein 4
DAPI: 4′-6-Diamidino-2-phenylindole
DC: Dendritic cells
DMEM: Dulbecco's modification of Eagle medium
DMSO: Dimethyl sulfoxide
ELISA: Enzyme-linked immunosorbent assay
FCS: Fetal calf serum
G-CSF: Granulocyte colony-stimulating factor
GI tumors: Gastrointestinal tumors
GROa: Growth-related protein alpha
IFNgamma: Interferon gamma
IL-10: Interleukin 10
ICH: Immunohistochemistry
IP-10: Interferon gamma-induced protein 10
I-TAC: Interferon-inducible T-cell alpha chemoattractant
LDH: Lactate dehydrogenase
(m): Membrane bound
MFI: Mean Fluorescence Intensity
MIP-1: Macrophage inflammatory protein
MSI: Microsatellite instability
MTT: 3-(4,5-Dimethylthiazol-2-yl)-2,5-diphenyltetrazolium bromide
PBMC: Peripheral blood mononuclear cells
PBS: Phosphate-buffered saline
PDL-1: Programmed death ligand 1
RPMI1640: Roswell Park Memorial Institute medium
(s): Soluble
SDF-1: Stromal cell-derived factor 1
sICAM-1: Soluble intercellular adhesion molecule-1
sTREM-1: Soluble triggering receptor expressed on myeloid cells-1
TNFalpha: Tumor necrosis factor alpha
VEGF: Vascular endothelial growth factor
