# Supplementary figures and images for: Induction of cytotoxic effector cells towards cholangiocellular, pancreatic, and colorectal tumor cells by activation of the immune checkpoint CD40/CD40L on dendritic cells
Source: Cancer Immunol Immunother. 2020 Nov 12;70(5):1451–64. doi: 10.1007/s00262-020-02746-x (PMC8053193; doi:10.1007/s00262-020-02746-x)

## Slide 1
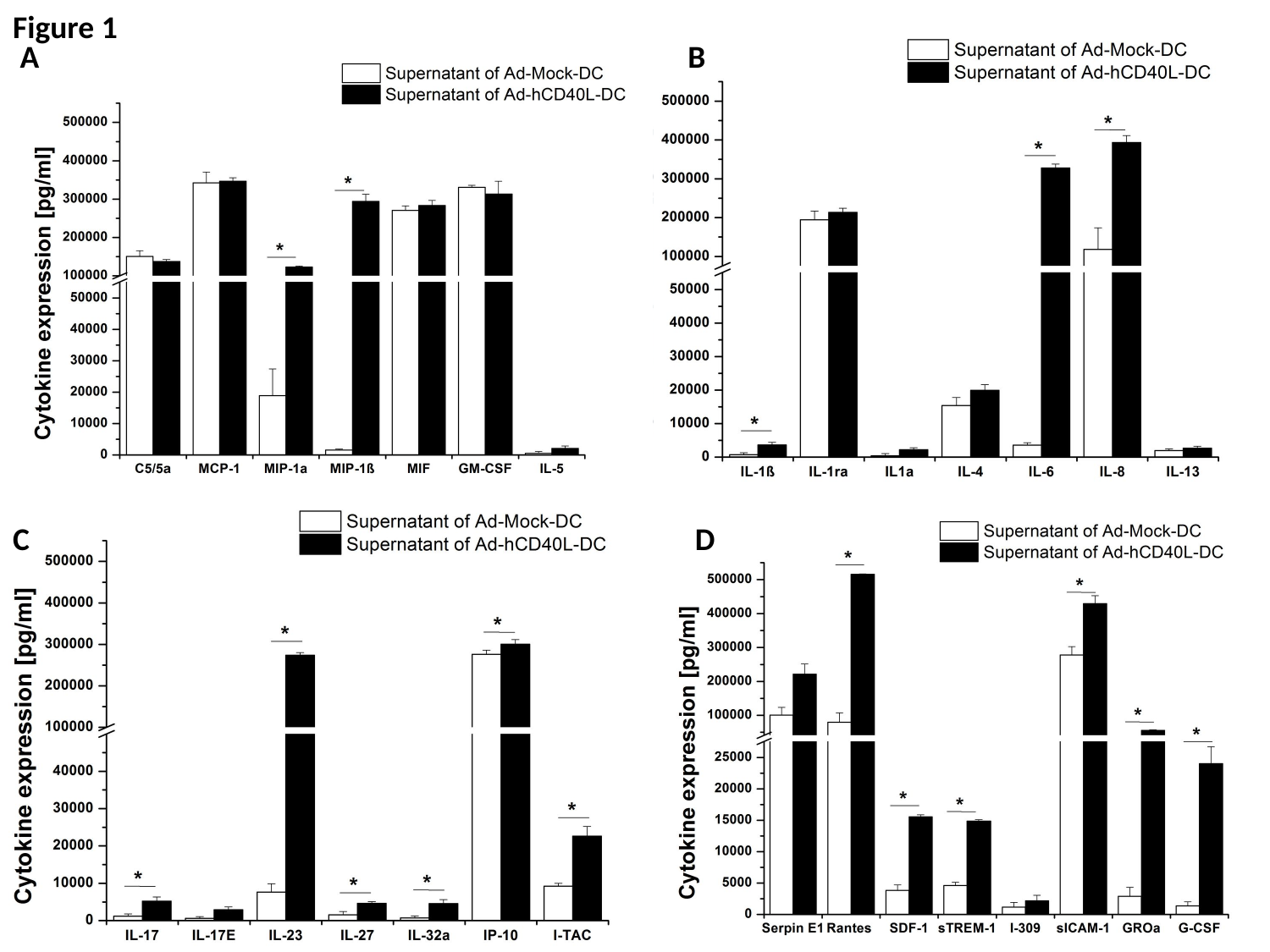

Figure 1
B
A
C
D

## Slide 2
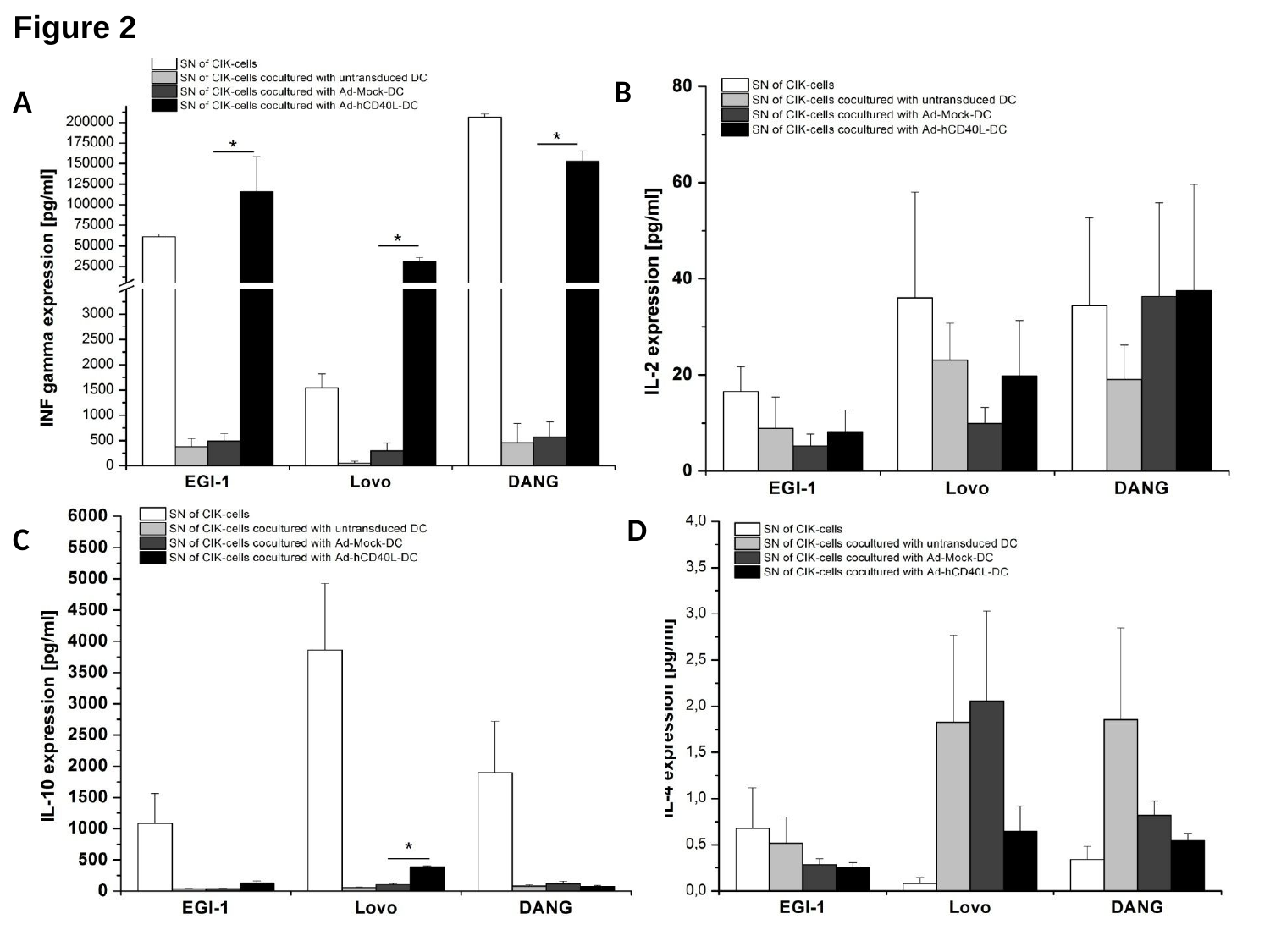

Figure 2
B
A
D
C

## Slide 3
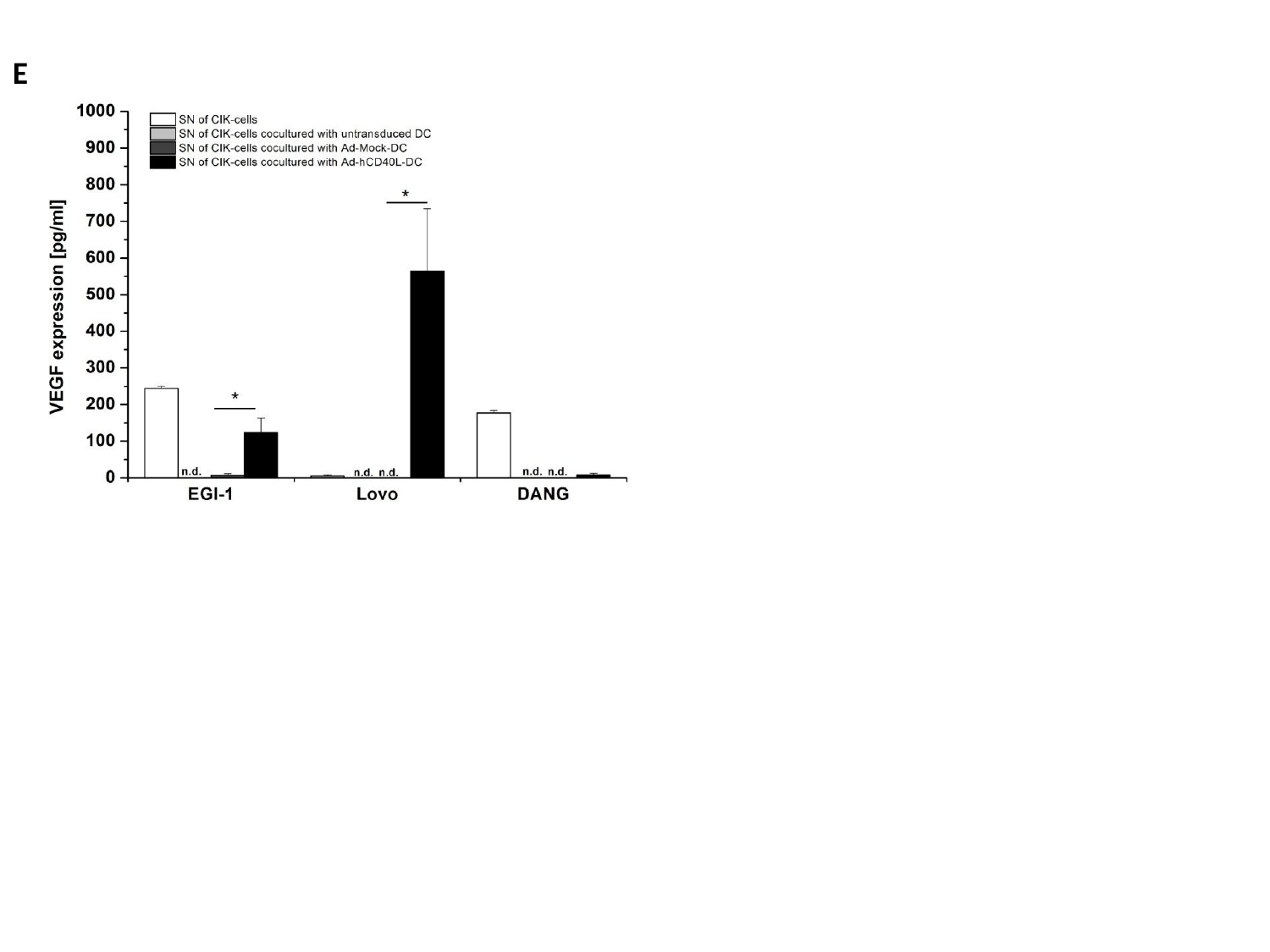

E

## Slide 4
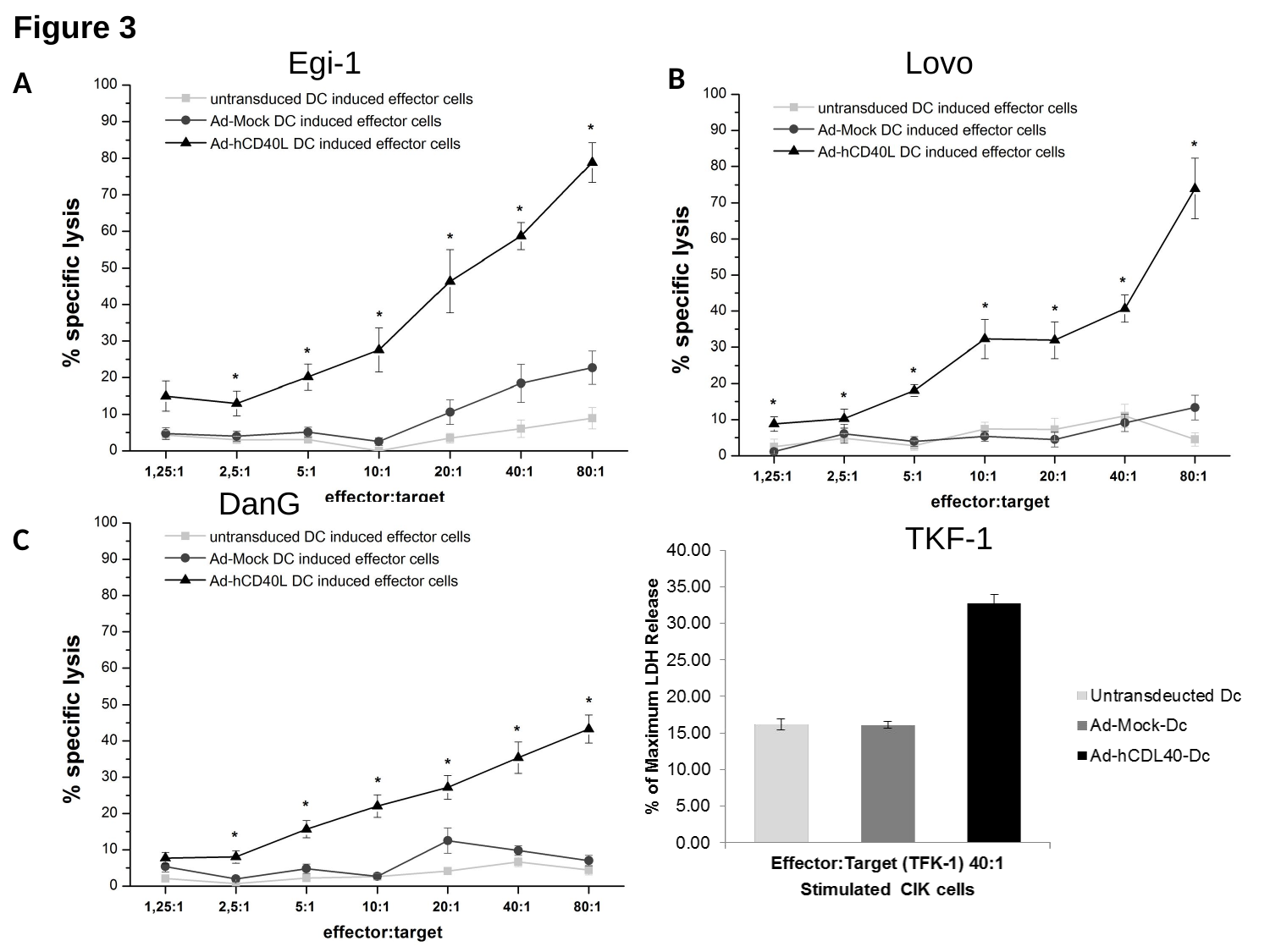

Figure 3
Egi-1
Lovo
B
A
DanG
TKF-1
C

Supplement: Supplementary file 1 — Figure 1 Cytokine/chemokine expression pattern after Ad-hCD40L transduction of DC using cytocine array.(A-D) Amounts of cytokine- and chemokine expression (pg/ml) in the supernatant of DC 48 hours after adenoviral transduction with Ad-hCD40L or Ad-Mock. n.d.=non detectable. Data represent means+/-SEM of four different experiments. (*=p<0.05). Figure 2 Cytokine expression in the supernatant of co-cultured effector cells with transduced DC.(A-E) Amounts of IFNγ (A), IL-2 (B), IL-10 (C), IL-4 (D) and VEGF (E) measured by ELISA in the supernatant of CIK cells cocultured with Ad-hCD40L-, Ad-Mock- or non-transduced DC for four days. DC were pulsed with tumor-lysate from -, EgI-1-, LoVo- and DanG-cells before coculture. Data represent means+/-SEM of three different experiments. (*=p<0.05). Figure 3 Enhanced cytotoxicity of effector (CIK) cells after cocultering with Ad-hCD40L transduced DC.Specific cytotoxicity of autologous effector cells (CIK-cells) towards, EgI-1-, Lovo- and DanG cells after coculture with Ad-hCD40L-, Ad-Mock- or non-transduced DC. Data represent means+/-SEM of three-four different experiments. (*=p<0.05). (PPT 1110 kb) [file 262_2020_2746_MOESM1_ESM.ppt]
